# HELLP syndrome complicated by ischemic colitis: A case report

**DOI:** 10.1002/ccr3.7557

**Published:** 2023-06-14

**Authors:** Paria Boustani, Laleh Eslamian, Maryam Nurzadeh, Vajiheh Marsosi, Marjan Ghaemi

**Affiliations:** ^1^ Student Research Committee Tehran University of Medical Sciences Tehran Iran; ^2^ Department of Obstetrics & Gynecology, Shariati Hospital Tehran University of Medical Sciences Tehran Iran; ^3^ Vali‐E‐Asr Reproductive Health Research Center, Family Health Research Institute Tehran University of Medical Sciences Tehran Iran

**Keywords:** HELLP syndrome, ischemic colitis, Microangiopathy, pregnancy, rectal bleeding

## Abstract

**Key Clinical Message:**

HELLP syndrome is a complicated disorder associated with many unknown complications, which ischemic colitis might be one of. Timely diagnosis and prompt management with multidisciplinary approach is the key for a favorable outcome.

**Abstract:**

HELLP syndrome is a triad of hemolysis (H), elevated liver enzymes (EL), and low platelet count (LP) which is considered a rare but serious pregnancy complication. HELLP syndrome is mainly associated with pre‐eclampsia, but can also occur individually. It may cause maternal and fetal mortality and some life‐threatening morbidity. The best management considered for HELLP syndrome is immediate delivery in most cases. We report a 32‐week pregnant woman with pre‐eclampsia who developed HELLP syndrome shortly after admission which led to a preterm cesarean section. Rectal bleeding and diarrhea started the day after delivery, and all the workups and imaging suggested ischemic colitis. She received intensive care and supportive management. The patient recovered and was discharged uneventfully. HELLP syndrome may be associated with many unknown complications, and ischemic colitis might be one of them. Timely diagnosis and prompt management with a multidisciplinary approach is the key to a favorable outcome.

## Graphical abstract

Histologic examination of colon biopsy suggestive of ischemic colitis in a patient diagnosed with HELLP syndrome.

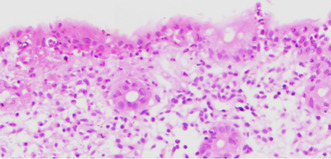

## 1 BACKGROUND

The HELLP syndrome is a triad of hemolysis (H), elevated liver enzymes (EL), and low platelet count (LP) which is considered a rare but serious pregnancy complication. HELLP can cause end‐organ failure and maternal and fetal mortality. Maternal mortality is reported between 1 and 30%. Morbidities like disseminated intravascular coagulation (DIC), eclampsia, hepatic damage, acute kidney injury, placental abruption, pulmonary edema, cerebral edema, preterm delivery, recurrent thrombosis, acute respiratory distress syndrome (ARDS), and sepsis are reported. HELLP syndrome is mainly associated with pre‐eclampsia, but can also occur individually. It mostly occurs between the 27th and 37th week of gestation, but 30% of cases are postpartum. The most common symptoms are right upper abdominal quadrant or epigastric pain, nausea, and vomiting. The best management considered for HELLP syndrome is immediate delivery, but expectant management can also be considered in some cases. Hypertension management and seizure prevention are also crucial.^1^, ^2^, ^3^ In this case report, we present a pregnant woman with HELLP syndrome complicated by ischemic colitis after a cesarean section.

## 2 CASE PRESENTATION

A 35‐year‐old gravid 1 pregnant woman with a gestational age of 32 weeks was admitted to the emergency room with a chief complaint of abdominal pain and proteinuria (430 mg/24 h) in her laboratory test results. She reported a history of elevated blood pressure (BP = 130/85 mm/hg) in the previous 2 weeks, but her vital signs were normal at the time of admission (BP = 120/80 mm/hg, PR = 80/min, RR = 13/min, T = 37°centigrade).

The physical examination only showed mild epigastric tenderness. Laboratory tests were requested; blood tests were normal but urine analysis confirmed proteinuria. The patient was admitted with a possible diagnosis of mild pre‐eclampsia.

On the second day of admission, she complained of severe persistent epigastric pain and her blood pressure increased to 160/100 mm/hg. Emergency delivery by cesarean section was performed with a diagnosis of severe pre‐eclampsia, and a girl with 1750 gram weight with Apgar 7–8 was born.

After delivery, the laboratory findings showed alanine aminotransferase (ALT) 1965 U/L, aspartate aminotransferase (AST) 2040 U/L, lactate dehydrogenase (LDH) 4485 U/L, and platelet count of 72 × 10^9/L, and peripheral blood smear revealed signs of hemolysis. The patient was diagnosed with HELLP syndrome and transferred to the intensive care unit (ICU).

The next day rectorrhagia and bloody diarrhea started with tachycardia (PR = 140/min) and fever (T = 38°centigrade) appeared. Basic supportive care such as hydration with normal saline was initiated. As rectal bleeding continued, hemoglobin dropped from 11 g/dL to 8 g/dL. 2 units of pack cell and 3 units of fresh frozen plasma (FFP) were transfused.

Based on the signs and symptoms, our differential diagnosis was infectious colitis, colitis due to vasculitis and ischemic colitis due to hypoperfusion; therefore, infectious disease, rheumatology, and gastroenterology consultation were requested.

According to the fever and consultation with an infectious disease specialist, 1250 mg Vancomycin BD and 2 g Meropenem TDS were administered. Stool analysis only showed blood. The stool and blood culture test results were normal.

Spiral abdominopelvic computed tomography (CT) scan (with and without intravenous and oral contrast) and abdominal aorta and pelvic CT angiography were suggested by a gastroenterology consultant, which reported multiple ischemic liver lesions (or liver infarcts), mucosal hyperenhancement and submucosal edema in ascending, transverse and descending colon, sigmoid and rectum with no evidence of active intraluminal extravasation of contrast; which was suggestive of colitis secondary to underlying microangiopathy.

**FIGURE 1:**
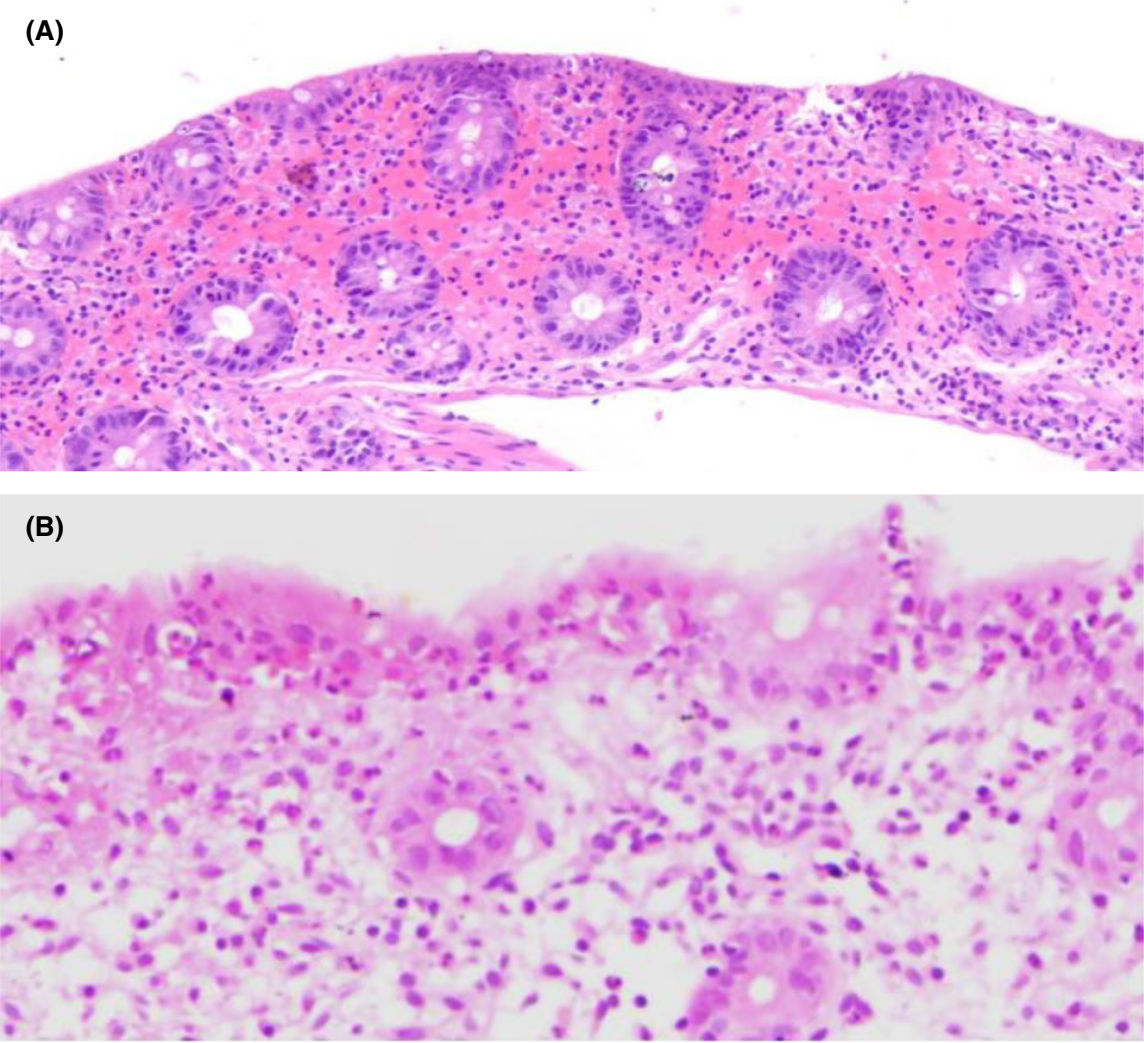
(A) Histologic examination of transverse colon biopsy showed relatively preserved architecture with foci of erosions and lamina propria hemorrhage and hyalinization. (HE stains, *100). (B) Histologic examination of ascending colon biopsy showed neutrophils infiltration in surface epithelium (highlighted by black arrowheads) and cryptitis (highlighted by white arrowheads). (HE stain, *400).

Endoscopy was normal but the colonoscopy suggested ischemic colitis or vasculitis; biopsies were also taken. The histomorphological findings of colon mucosal biopsy showed mild lamina propria neutrophil infiltration and multifocal surface erosions in ascending and transverse colon. Portions of colonic mucosa with an active colitis pattern in the descending colon were noted, which was suggestive of ischemic colitis (Figure 1A,B).

The blood tests requested by the rheumatology consultant were all normal, and there were no signs of hypotension; therefore, vasculitis and hypoperfusion were ruled out. It seems like ischemic colitis was an unusual complication of the HELLP syndrome in this case.

On the tenth day of admission, all the symptoms and laboratory findings were resolved. The patient was discharged in good general condition. Follow‐up 1 month later showed no recurrence of gastrointestinal (GI) bleeding.

## 3 DISCUSSION

HELLP syndrome (hemolysis, elevated liver enzymes, low platelet count) is a life‐threatening pregnancy complication with unknown etiology, which mainly occurs in patients with hypertension and pre‐eclampsia. Although the etiology is not clear, it is thought to be a systemic inflammatory disorder associated with impaired placentation, complement activation, and coagulation cascade. There is an increased inflammatory response in HELLP syndrome, rather than in normal pregnancies.^4^, ^5^

Placental ischemia causes maternal vascular endothelium activation, leading to increased anti‐angiogenic factors. These anti‐angiogenic factors cause systemic endothelial dysfunction. Platelet adhesion on damaged endothelium activates a coagulation cascade; leading to vasospasm, platelet aggregation, and more endothelial damage; resulting in thrombocytopenia. Red blood cells passing through capillaries with damaged endothelium enriched with platelet and fibrin strands lead to microangiopathic hemolytic anemia (MAHA). Microvascular fibrin deposits cause obstruction in hepatic sinusoids, leading to hepatic blood flow obstruction and liver dysfunction, causing elevated liver enzymes. These events explain the classic triad of HELLP syndrome.^4^, ^5^, ^6^

Systemic endothelial damage, complement dysregulation, and high serum levels of active multimeric von will brand factor (VWF) result in thrombotic microangiopathy and multiorgan microvascular injury seen in HELLP patients, which can involve the mesenteric vasculature.^4^, ^5^

Ischemic colitis is a result of insufficient blood supply to the colon. Acute changes in the systemic circulation or the local mesenteric vasculature result in not meeting the metabolic demands of the colon, causing mucosal inflammation. Mucosal edema followed by the involvement of submucosal arterioles causes ulceration and hemorrhage. Common symptoms of ischemic colitis are diarrhea and rectal bleeding, just like our patient.^7^, ^8^, ^9^

**FIGURE 2:**
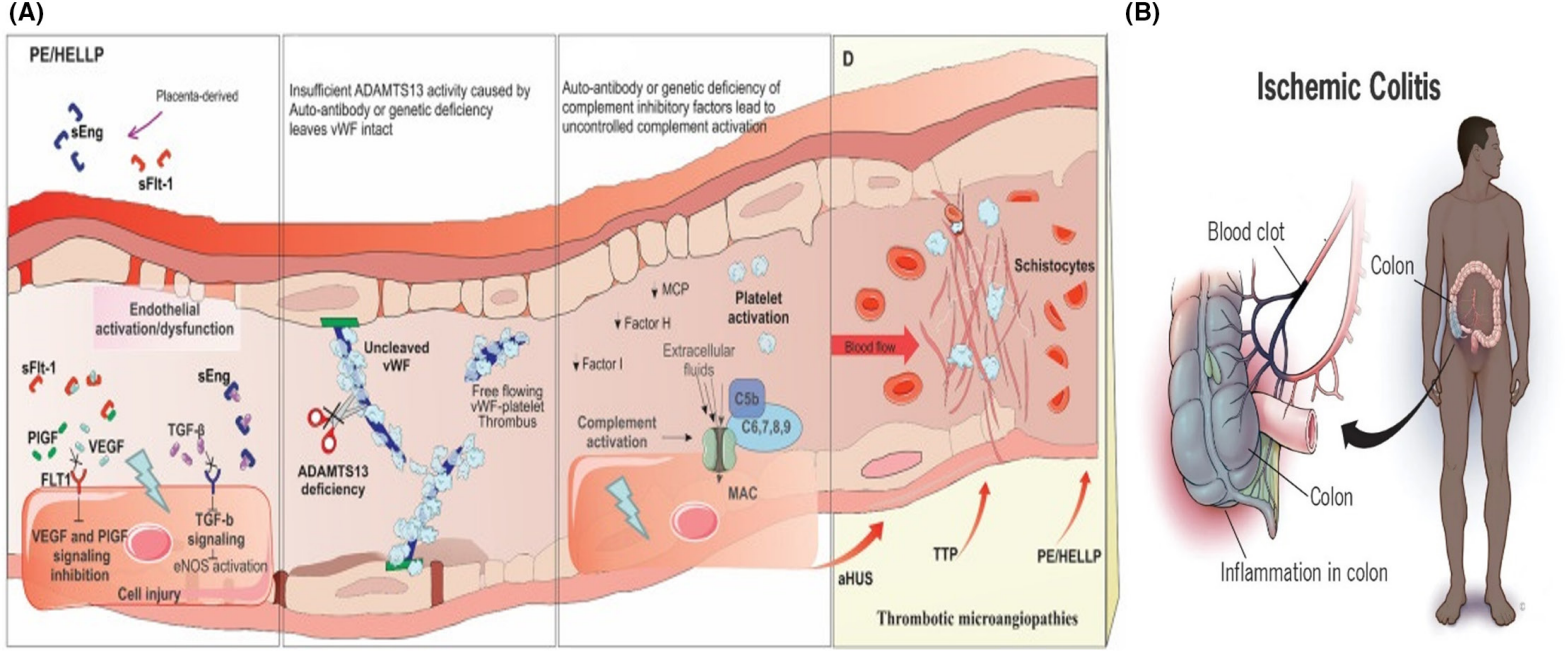
(A) Pathophysiology of thrombotic microangiopathy in HELLP syndrome^10^. (B) Ischemic Colitis pathophysiology.^11^ Thrombotic microangiopathy in HELLP syndrome may lead to blood clot formation in the mesenteric vasculature, causing ischemic colitis.

Systemic circulation alterations and involvement of the mesenteric vasculature due to thrombotic microangiopathy and multiorgan microvascular injury seen in HELLP patients might be the underlying cause of ischemic colitis in this case (Figure 2A,B).^10^, ^11^

Our case report presents a very rare case of ischemic colitis followed by HELLP syndrome. Our patient had all the criteria for HELLP syndrome and received intensive care and supportive management, but rectal bleeding was an unpredictable complication. CT imaging and multiphasic CT angiography (CTA) followed by colonoscopy suggested ischemic colitis. She received medical management with intravenous fluid resuscitation, blood transfusion, bowel rest, and intravenous antibiotics. Following effective teamwork, our patient recovered uneventfully.

## 4 CONCLUSION

HELLP syndrome is a complicated disorder associated with many unknown complications, of which ischemic colitis might be one. Timely diagnosis and prompt management with a multidisciplinary approach is the key to a favorable outcome.

## AUTHOR CONTRIBUTIONS

**Paria Boustani:** Data curation; writing – original draft; writing – review and editing. **laleh eslamian:** Conceptualization; supervision. **maryam nurzadeh:** Supervision; validation. **vajiheh marsosi:** Supervision. **marjan ghaemi:** Supervision; writing – review and editing.

## FUNDING INFORMATION

None.

## CONFLICT OF INTEREST STATEMENT

None.

## ETHICS STATEMENT

The patient signed the informed consent. This study was performed according to Helsinki deceleration and the identity of the participant is confidential.

## CONSENT

Written informed consent was obtained from the patient for publication of this case report and accompanying images. A copy of the written consent is available for review by the editor in chief of this journal on request.

## Data Availability

Data supporting the findings of this study are available from the corresponding author, upon reasonable request.

## References

[1] Adorno M , Maher‐Griffiths C , Grush Abadie HR . HELLP Syndrome. Crit Care Nurs Clin North Am. 2022;34(3):277‐288. doi:10.1016/j.cnc.2022.04.009 36049847

[2] Dusse LM , Alpoim PN , Silva JT , Rios DR , Brandão AH , Cabral AC . Revisiting HELLP syndrome. Clin Chim Acta. 2015;451:117‐120. doi:10.1016/j.cca.2015.10.024 26525965

[3] Haram K , Svendsen E , Abildgaard U . The HELLP syndrome: clinical issues and management. A Review. BMC Pregnancy Childbirth. 2009;9:8. doi:10.1186/1471-2393-9-8 19245695 PMC2654858

[4] Petca A , Miron BC , Pacu I , et al. HELLP syndrome‐holistic insight into pathophysiology. Medicina (Kaunas). 2022;58:326. doi:10.3390/medicina58020326 35208649 PMC8875732

[5] Khalid F , Mahendraker N , Tonismae T . HELLP Syndrome. StatPearls [Internet]. StatPearls Publishing; 2022. 32809450

[6] Padden MO . HELLP syndrome: recognition and perinatal management. Am Fam Physician. 1999;60(3):829‐836. 10498110

[7] Hung A , Calderbank T , Samaan MA , Plumb AA , Webster G . Ischaemic colitis: practical challenges and evidence‐based recommendations for management. Frontline Gastroenterol. 2019;12:44‐52. doi:10.1136/flgastro-2019-101204 33489068 PMC7802492

[8] Theodoropoulou A , Koutroubakis IE . Ischemic colitis: clinical practice in diagnosis and treatment. World J Gastroenterol. 2008;14(48):7302‐7308. doi:10.3748/wjg.14.7302 19109863 PMC2778113

[9] Komeno Y , Ogawa S , Ishida T , et al. Ischemic colitis as a manifestation of thrombotic microangiopathy following bone marrow transplantation. Intern Med. 2003;42(12):1228‐1232. doi:10.2169/internalmedicine.42.1228 14714965

[10] Zununi Vahed S , Rahbar Saadat Y , Ardalan M . Thrombotic microangiopathy during pregnancy. Microvasc Res. 2021;138:104226. doi:10.1016/j.mvr.2021.104226 34252400

[11] Ischemic Colitis article by Cleveland Clinic. [Available from: https://my.clevelandclinic.org/health/diseases/24513‐ischemic‐colitis]

